# Pediatric Preformed Zirconium Oxide Crowns vs. Preformed Metal Crowns After Pulpotomy in Primary Molars: A Practice-Based Retrospective 2.5 Year Cohort Study

**DOI:** 10.3390/healthcare13070751

**Published:** 2025-03-27

**Authors:** Isabella Brenner, Maria Abdin, Julian Schmoeckel

**Affiliations:** 1 Zahnärztehaus Smile & Little Smile, 77871 Renchen, Germany; 2 Department of Pediatric Dentistry, University Medicine of Greifswald (UMG), 17475 Greifswald, Germany; maria.abdin@uni-greifswald.de

**Keywords:** pulpotomy, primary molars, stainless steel crowns, zirconia crown

## Abstract

**Background:** Primary molars with deep carious lesions often require a treatment with pulpotomy and restoration with a crown. **Aim:** This study aims to compare the survival rates of stainless steel (SSC) and zirconium oxide (ZOC) crowns carried out on pulpotomized primary molars using the International Caries Detection and Assessment System (ICDAS) 4 to 6 lesions. **Materials and Methods:** The data of 100 patients (mean age 5.3 ± 2.1 years, mean decayed, missing or filled primary teeth (dmft) 7.1 ± 3.2) with 272 primary molars (40, 225, 7 with ICDAS 4, 5, 6, respectively) were collected retrospectively from a specialized private pediatric dental office after ethical approval was obtained and each participant agreed to participation in the study. Primary molars were treated between 2019 and 2021 with pulpotomy (15.5% ferrous sulfate solution for hemostasis and zinc oxide eugenol as a wound dressing) followed by a crown (203 SSC and 69 ZOC) with a minimum follow-up period of 6 months and a mean follow-up time of 28.2 (±11.0) months. **Results:** Failure occurred significantly less often in SSC (n = 13 out of 203) than in ZOC (n = 20 out of 69; *p* < 0.001). Major failure was attributed to swelling and abscess (n = 13, 39.4%) followed by the occurrence of fistula (n = 15, 45.4%) and fracture of the crown and abutment (n = 5, 15.2%). Minor failure due to cement dissolution occurred significantly less often in SSC than in ZOC (n = 10 out of 203 vs. n = 9 out of 69; *p* < 0.005. A Kaplan–Meier survival analysis showed an overall estimated survival time of 38.25 (Confidence interval (CI): 37.0–39.4) months for both types of crowns. A Log-Rank (Mantel–Cox) analysis showed a statistically significant difference (*p* < 0.05) in the estimated mean survival time of SSC (39.75; CI: 38.5–40.9 months) and ZOC (33.4; CI: 30.5–36.3 months). Survival probability drops just below 80% for ZOC and stays a little over 90% for SCC around the 20th month. **Conclusions:** SSC showed an advantage over its ZOC counterpart when placed after pulpotomy for the management of primary molars with deep carious lesions. A higher necessity for re-intervention in the more aesthetic ZOC should be considered in clinical decision taking.

## 1. Introduction

Pulpotomy is a treatment measure performed on symptom-free teeth in the case of extensive carious lesions, i.e., when the pulp in the carious dentin is exposed during caries removal or tooth preparation [1]. The aim of pulpotomy is to preserve the vitality of the radicular pulp and the formation of hard tissue between that remaining pulp and the wound dressing. An X-ray should be taken to rule out pathological resorption and periradicular inflammation. After excavation of the carious lesion and exposure of the pulp, the coronal pulp is removed. This step is usually carried out using rotary instruments and water cooling. Hemostasis can be performed with a variety of materials such as saline, ferrous sulfate or, earlier, with formocresol [2,3]. The formation of a blood clot at the amputation site should be avoided at all costs. Suitable wound dressings include mineral trioxide aggregates (MTA) or zinc oxide eugenol cement [1,4]. The pulpotomized tooth should then be restored with a pediatric crown for stability, either a stainless steel crown (SSC) or a zirconium oxide crown (ZOC) [1].

The literature on the success and the impact of aesthetic crowns after pulp treatment is scarce [5], while SSCs have been widely used and investigated [6,7]. The topic of post-pulpotomy restoration is a frequent issue in daily specialized pedodontics service and needs more investigation. In clinical decision making, general aspects, such as cost coverage issues, time efficiency, treatment modality, and aesthetic concerns, play an important role aside from the tooth-level aspects of when and how to intervene in the carious process [8,9]. Consequently, the question arose as to whether the restoration of a pulpotomized deciduous molar with a ZOC is an equivalent alternative to the restoration with an SSC. In the literature, there is no randomized controlled trial investigating this question. The only recent study with a similar approach to this project was also a comparison of ZOC and SSC after pulpotomy, in a different private practice in Germany, showing similar success rates after about 1.5 years with a tendency for better outcomes in SSCs [7]. This research addresses the knowledge gap regarding the impact of the post-pulpotomy restoration in primary molars. Research on ZOC is usually performed on anterior teeth after ECC [8,9]. To date, studies comparing SSC and ZOC for primary molars are unfortunately still scarce and usually inherit methodological limitations like selection bias and recollection bias within their frameworks. We tried to minimize this in this project by applying a retrospective evaluation of performed treatment (e.g., real-life) but a prospective evaluation regarding the outcome (e.g., precise documentation). Randomized controlled trials are often desirable but, to our knowledge, not yet available on this topic.

Therefore, the aim of this study was to retrospectively determine the survival rates of stainless steel (SSC) and zirconium oxide (ZOC) crowns carried out on pulpotomized primary molars with 15.5% ferrous sulphate solution for hemostasis and zinc oxide eugenol cement as a wound dressing in a private practice setting.

## 2. Materials and Methods

### 2.1. Ethical Aspect

The written ethical approval for this retrospective cohort study was obtained on 22.01.2022 prior to data collection from the ethics committee of the University Medicine Greifswald (BB 193/21). The study was performed in accordance with the declaration of Helsinki and GCP guidelines. All participants/legal guardians gave written consent for study participation at a recall appointment and filled out a questionnaire on the acceptance of the crown in that scheduled dental visit, at the private practice “Zahnärztehaus Smile & Little Smile”, located in Renchen, Germany.

### 2.2. Study Design

The aim of this study is to present retrospectively collected data from patients who received a pulpotomy between 1 January 2019 and 31 December 2021 in the form of an index card study of patients who underwent pulpotomy of the deciduous molar in a German private practice. The study participants were between 1 and 10 years old at the time of the treatment. The ICDAS index (International Caries Detection and Assessment System) was used for visual caries diagnosis [10]. Radiographic assessment of caries was based on ADA criteria [11].

The inclusion criteria included:

- Children aged 1–10 years old, who have received a ZOE pulpotomy and a subsequent restoration of the primary molar with a pediatric stainless steel crown (SSC from 3M ESPE) or zirconium crown (NuSmile) in the time frame 01.01.2019 to 31.12.2021.
- Minimum follow-up appointment in the digital dental records of 6 months with the last day of considering a follow-up appointment being 22.12.2023.
- Informed and written consent to participate in the study by the legal guardian, obtained at any of the recall appointments.
- Completion of a questionnaire about the satisfaction and acceptance of the crown.

**Figure 1:**
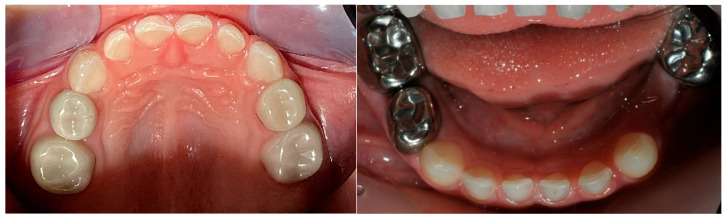
Examples of zirconium crowns and stainless steel crowns in situ during recall appointment of patients participating in the study. (photos: courtesy of I. Bre).

The treatments were performed either under general anesthesia (DGA: dental general anesthesia), with local anesthesia under nitrous oxide sedation, or chairside only with local anesthesia without the intent to be scientifically evaluated, making the treatment a real-world-setting. Relative moisture control was always provided by cotton rolls and constant suction. Pulpotomy was performed with zinc oxide eugenol (ZOE) special paste (normal-curing) from Speiko (Speiko GmbH, Bielefeld, North Rhine-Westphalia, Germany) and 15.5% ferrous sulphate solution (Astringedent^®^, Ultradent Products GmbH, Cologne, North Rhine-Westphalia, Germany). Subsequently, the deciduous molar was prepared minimally, proximally, and occlusally (approx. 1 mm) with high-speed bur to accommodate a stainless steel crown, or invasively (approx. 2 mm) on all 5 surfaces of the tooth to accommodate a zirconium oxide crown. Stainless steel crowns with a bulbous curvature to allow a “snap” over the deciduous tooth to increase retention were used (3M Espe AG, Landsberg am Lech, Bavaria, Germany). The stainless steel crowns were cemented with glass ionomer cement (Ketac cem plus; 3M Espe AG, Landsberg am Lech, Bavaria, Germany) (Figure 1). The zirconium crowns used were NuSmile Zirconia Crowns (NuSmile, Houston, TX, USA) which were cemented with RelyX (3M Espe AG, Landsberg am Lech, Bavaria, Germany) (Figure 1). RelyX is a self-adhesive and dual-curing composite cement.

The decision on the type of crown, stainless steel crown or zirconia crown, to restore the pulpotomized teeth was made jointly with the parents according to routine practice procedure. The parents were informed before the treatment that the restoration of the pulpotomized deciduous molar with a stainless steel crown was the most economical, appropriate, and evidence-based treatment and that the costs would be covered by the public health insurance. Treatment with a zirconium oxide crown goes beyond cost-effectiveness and is an optional service. Private billing was carried out by a billing company, which is why the parents were offered payment in installments. In addition, the parents were informed that extensive grinding of the deciduous molar to accommodate a zirconia crown may be associated with an increased risk of loss of retention of the pediatric crown, as well as fistula and abscess formation.

The participants were followed up independently of the study depending on their individual caries risk. Recalls were set at 3- or 6-month intervals and any complaints concerning the treated teeth or the loss of the pediatric crown were documented and available for retrospective analysis. The parents were asked to participate in the study at a follow-up appointment; in that way the outcomes were evaluated prospectively and not from the routine patient documentation, which often lacks important information. Upon consent, parents were given a questionnaire to fill out directly. The aim of the questionnaire was to assess the acceptance of the therapy.

### 2.3. Evaluation of Treatment

The pulpotomized deciduous molar with a pediatric crown was evaluated prospectively after informed consent and considered to survive if it was asymptomatic in situ and fulfilled its function. It was considered successful if it exfoliated physiologically with the eruption of the permanent tooth. Crown loss of the pediatric crown, where the pediatric crown could be re-cemented after cleaning and removal of cement residue, was considered a minor failure. The following events were considered major failures:

- The pulpotomized primary molar with pediatric crown could not regain its function.
- Indication for, or performed, pulpectomy or extraction of the pulpotomized molar.
- Severe gingival inflammation or swelling, e.g., gingival/sub-mucosal abscess, which led to the removal of the pulpotomized molar.
- Fracture of the clinical dental crown with pediatric crown, which led to the extraction of the pulpotomized tooth with pediatric crown.

**Figure 2:**
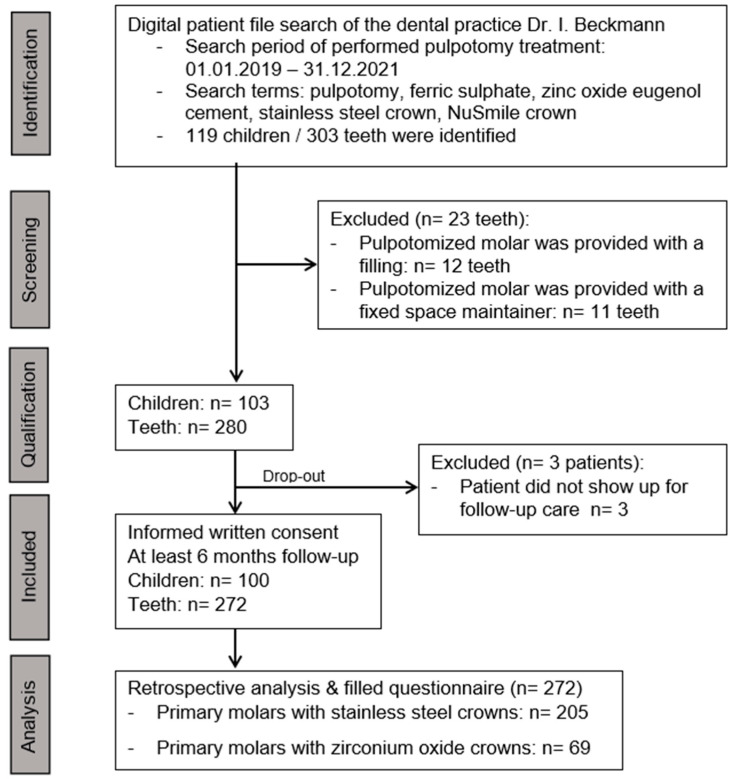
Flow chart of the study from participant identification to analysis.

Moreso, adherence to this private practice was very high as children in the recall system receive reminders before their appointments, which leads to a very small number of dropouts (Figure 2) and increases the validity of the data.

### 2.4. Questionnaire on Acceptance of the Treatment

Another aim of this study was to determine the acceptance and evaluation of the treatment, as well as the parents’ satisfaction, using a questionnaire. The questionnaire was filled out by the accompanying parent at a follow-up appointment. The patient had already been treated at the time of consent for participation in the study along with filling out the questionnaire. The questionnaire aimed at better assessing the child’s behavior and the child’s and parental acceptance of the therapy, for example with the Visual Analogue Scale (VAS scale) for fear before and after the treatment, pain after the treatment, or the general acceptance of the pediatric crowns.

### 2.5. Data Collection and Analysis

Initially, the data was collected in pseudo-anonymized form and the descriptive, retrospective data analysis was carried out in Excel (Microsoft Office Excel 2019, Microsoft Corporation, Redmond, WA, USA). The statistical analysis of the data was carried out both in Excel and in statistical software (SPSS Version 26 for Windows, Inc., Chicago, IL, USA). Numerical data were presented using means and standard deviations. Categorical data were presented as frequencies and percentages. T-test for Equality of Means and Leven’s Test for Equality of Variance were used to compare means. For analytical evaluation, a Chi-square test was performed in the case of categorical data. Kaplan–Meier survival analysis was carried out. Cross tables are used to better show the impact of multiple variables on the outcome. The threshold for statistical significance was defined as *p* < 0.05. A total of 272 pulpotomized deciduous molars with subsequent restoration with a stainless steel crown (n = 203) or zirconia crown (n = 69) in 100 children were included in the study.

## 3. Results

### 3.1. General Characteristics

The 57 boys and 43 girls were, on average, 5.3 (±2.1) years old on the day of the treatment with pulpotomy and crown and had a mean caries experience of 7.1 (±3.2) dmft (Table 1). The average follow-up time was 28.22 (±11.0) months with no significant difference between SSC and ZOC (*p* > 0.05; Table 2). Most (81.6%) of the ZOE pulpotomy and crown treatment was performed under general anesthesia, 16.5% under nitrous oxide sedation with local anesthesia, and 1.8% under local anesthesia only. No zirconium crowns were placed under local anesthesia and only one zirconium crown was placed under nitrous oxide sedation (Table 2).

### 3.2. Survival and Failure Rate of the Treatment

**Figure 3:**
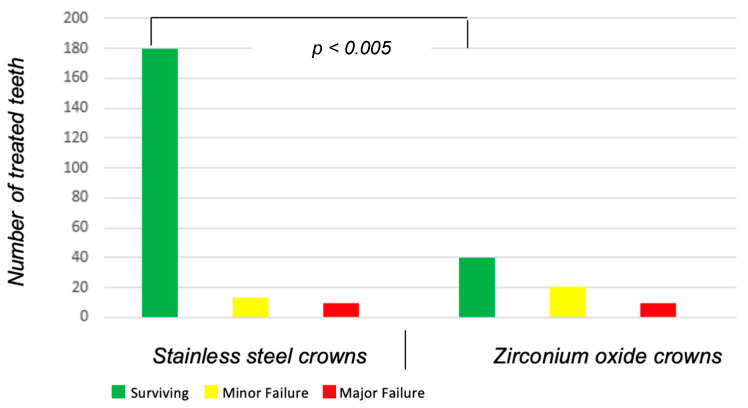
Evaluation of the treatment of pulpotomized deciduous molars with stainless steel crowns (bars 1–3 on the left) and zirconium oxide crowns (bars 4–6 on the right). The survival rate of the stainless steel crowns is statistically significantly higher than that of the zirconium oxide crowns (*p*-value < 0.005). The odds ratio of SSC vs. ZOC is 5.9 (95% CI: 2.7–12.8).

The stainless steel crown was statistically significantly more successful than the zirconium crown (*p* < 0.005) in the retrospective evaluation with almost a mean of 2.5 years follow-up. The failure rates were statistically significantly (*p* = 0.018) and clinically relevantly different along with the statistically significantly different mean survival duration (SSC: 31.4 months vs. ZOC: 23.4 months; Table 3). Overall, 33 crowns had major failure: 13 out of 203 stainless steel crowns (6.4%) and 20 out of 69 (29.0%) zirconia crowns (Table 4 and Figure 3). The odds ratio in relation to the treatment of pulpotomized deciduous molars with subsequent restoration with a stainless steel against a zirconia crown is 5.9 (95% CI: 2.7–12.8). The probability of clinical failure is therefore 5.9 times higher when a zirconia crown is applied. Still, most of the treated teeth (87.9%) were asymptomatic (successful and minor failure). Frequent reasons for minor failure were cement dissolution, whereby re-cementation of the pediatric crown was possible (n = 19, 7.0%). The most frequent reason for major failure was swelling or abscess followed by the occurrence of fistulas and fracture of the clinical crown with pediatric crown (Table 4). Fifteen crowns failed within the first year of insertion, nine of which were SSC. ZOC had more failures in the following year.

### 3.3. Survival Analysis

**Figure 4:**
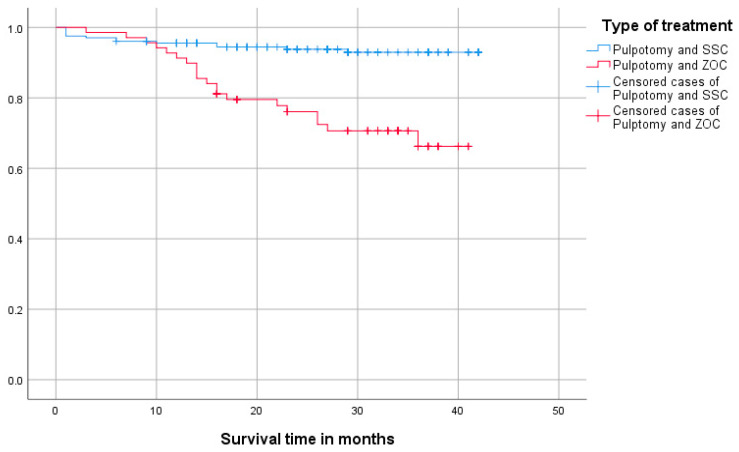
Kaplan–Meier survival plot of stainless steel (SSC) and zirconium oxide crowns (ZOC).

Overall, the cumulative survival time was 38.25 (CI: 37.0–39.4) months for both types of crowns; 39.75 (CI: 38.5–40.9) months for SSC and 33.4 (CI: 30.5–36.3) months for ZOC with a statistically significant difference between both groups (*p* < 0.001). Figure 4 shows the Kaplan–Meier survival plot of each type of crown. At the 20 months marker, survival probability drops just below 80% for ZOC and stays a little over 90% for SCC.

Interestingly, though the size of subsamples is partially very low, the failure rates for SSC under nitrous oxide sedation (15.9%) were higher than for SSC under DGA (3.9%). At the same time, the failure rate of ZOC with DGA was clearly higher (29.4%) even when compared to SSC under N_2_O-sedation (Table 5), despite the assumed better working conditions under DGA.

### 3.4. Acceptance of the Treatment with Pediatric Crowns

The acceptance of stainless steel crowns or zirconia crowns in situ was very high. Only the legal guardians of two girls and two boys made negative comments about their nine stainless steel crowns due to the aesthetics. Patients who received a zirconium oxide crown were exclusively positive as there were no negative comments about the zirconia crowns in the questionnaire. Overall, the crowns were well accepted (96.7%). The stainless steel crowns were rated positively by 95.6% of the parents, revealing a very high subjective satisfaction with both treatment modalities.

## 4. Discussion

### 4.1. Treatment Evaluation

The survival rates of performed crowns placed on pulpotomized deciduous molars with ferrous sulphate and zinc oxide eugenol cement in the present study are in line with those of the current literature [12,13,14]. Recently, MTA cements or biodentine are more recommended as wound dressings for pulpotomy [1,15,16]. Overall, it can be assumed that the success rate for treatments with MTA cements is somewhat higher than with zinc oxide eugenol cement [17]. Therefore, there might have been slightly fewer (major) failures if a more modern wound dressing had been used for pulpotomy in this sample of patients. However, these materials are more expensive than zinc oxide eugenol cements and may not be covered by dental insurance in Germany. The restoration with a zirconia crown is more costly [18] and might be less cost-effective. Since it is an optional service outside of health insurance coverage in Germany, the costs must be paid privately by the patients/parents.

The higher failure rate of zirconia crowns can probably be attributed to several reasons: (1) Tooth preparation; (2) Needed patient compliance and cooperation/time; (3) Cementation technique and retention. The pulpotomized deciduous molar requires more tooth preparation as the zirconia crown has a higher material thickness than the stainless steel crown. This results in a reduced height that leads to a reduced bonding surface, or an enlarged marginal gap may occur. The tooth preparation should also ideally be equi- or subgingival, which makes the needed moisture control for the sensitive cementation technique more difficult and more prone to failure [19]. Furthermore, the stainless steel crown can be adapted to the pulpotomized deciduous molars by bending the edge and can be seated quickly even when using a bioactive calcium silicate cement [20]. This increases the retention of the stainless steel crown and allows a better marginal fit via a so-called “snap fit” leading to a generally superior clinical performance for the restoration of primary molars [21] and reduces working time [20]. Due to the material properties of the zirconia crown, this is not possible, and the amount of retention might depend more on the practitioner’s experience and skill, which may be reflected by the decementation rates. This shows that time and experience needed for tooth preparation, costs, and technique sensitivity may impact the use of ZOC in daily practice [19].

Interestingly, another similar practice-based study only found a slightly lower success rate for ZOC than SSC which was not statistically significantly different [7] in contrast to the data of this study. Moreso, a study from Australia in which ZOC were solely placed under GA found good mid- to long-term survival rates [19]. However, in this study also, only one operator performed the treatment, and it may still be accounted true that the procedure is more complicated and the application of zirconium crowns ultimately more prone to error, even under the most optimal conditions like general anesthesia (Table 2). This leads to the conclusion that, for a more widespread use, ZOC may not to be recommended as the first choice. Ultimately, all studies show similar success or failure rates for pulpotomy itself with differences regarding the material used as a wound dressing [12,13,16].

### 4.2. Survival Rates of SSC vs. ZOC

The statistically significant difference in mean survival time between ZOC and SSC should be considered clinically relevant (23 vs. 31 months). This is especially true for young children with either only primary dentition (48% of the sample) or up to the beginning of mixed dentition (41% of the sample), as an invasive re-intervention like an extraction (major failure) may undergo the risk of needing another DGA. Moreso, even the recementation of a crown (minor failure) may inherit this risk. To clarify, there is a difference between survival and success as teeth with minor failure may still survive after reinterventions like a recementation.

Regarding the type of post-pulpotomy restoration, the survival rate for stainless steel crowns under nitrous oxide sedation with local anesthesia was 37 out of 44 (Table 5) and even better than for zirconia crowns under DGA (70.6%). A clear statement on the success or failure rate of pulpotomized deciduous molars under local anesthesia is not possible, as the number of teeth was too small. The same applies to treatments with zirconia crowns under nitrous oxide sedation with local anesthesia. Under general anesthesia, however, the success rate for stainless steel crowns was the highest at 96.1%. This shows that the failure rate of pulpotomized deciduous molars with stainless steel crowns under nitrous oxide sedation along with local anesthesia (16%) was higher than that under general anesthesia. This can be explained by the fact that the decision to treat a high-risk tooth under nitrous oxide sedation is more likely to be made than treatment under general anesthesia, where the tooth would then be extracted. Furthermore, almost all treatments of pulpotomized deciduous molars with zirconia crowns were performed under general anesthesia. With this type of treatment, the child’s cooperation during the treatment is irrelevant. It provides an ideal working environment for the practitioner but does by far not guarantee 100% success, as frequently a second DGA is needed for restorative or surgical re-intervention [22]. Still, the main outcome of this research shows that the survival rate of pulpotomized deciduous molars with zirconia crowns was lower than that of stainless steel crowns. Interestingly, the zirconia crown under general anesthesia has an even lower success rate than the stainless steel crown under nitrous oxide sedation. This underlines the fact that the ZOC procedure must be more difficult and prone to error, reflected by the data of this study but in contrast to data from another prior study [23].

Looking critically at the sample sizes of this research, an unequal number between ZOC and SSC can be observed, which is to be expected as real-world retrospective clinical data almost never have equal numbers in both groups, not even always in RCTs. Generally, larger differences in sample sizes may limit the validity of the statistical analysis and potentially the applicability of the findings. Still, due to high differences in outcome, the relatively smaller sample size of ZOC of 69 is still reasonably large and sufficient for statistically significant findings. Moreso, a follow-up period of 26.7 months is unique and beyond the minimum desired time span of 2 years for retrospective studies.

Additional general and patient-related factors could have been documented as they may influence the failure rates of different types of performed crowns. Still, to achieve causality between the failure of a performed crown, for example due to bad oral hygiene, high sugar intake, and no fluoride exposure, is clinically difficult to attribute. Based on the retrospective nature of the study with a prospective outcome evaluation, the authors opted for more local factors as a source of risk of failure. We believe this study offers important data on the survival rates of SSC and ZOC in pulpotomized primary molars, reinforcing the clinical advantage of SSC in terms of durability and lower failure rates. However, the study’s partial retrospective nature, sample imbalance, and lack of control for confounding factors limit the generalizability of its findings. Future prospective studies with randomized controlled designs are desirable and even longer follow-up periods up to about 5–7 years would strengthen the conclusions drawn from this research.

### 4.3. Acceptance

Before starting treatment, parents should be informed in detail about the type of pediatric crown (stainless steel crown or zirconia crown) and its advantages and disadvantages. It is essential to weigh up whether the better aesthetics and high acceptance of the zirconia crown [24] outweigh the increased risk of its failure. Restoring pulpotomized deciduous molars with a stainless steel crown is probably the only option until the deciduous molar exfoliates physiologically. This may have a positive effect on the child’s behavior at the dentist’s and is also in the overall context of improving children’s oral health. Another study also found similarly high parental satisfaction with both crown types, while the color acceptance was higher for ZOC [25].

The acceptance of pedodontic crowns can also be easily determined months after treatment, as the children can and could be asked and involved directly showing a very high acceptance. The sole reason for the negative evaluation of the stainless steel crown was not a lack of accuracy of fit or suitability for everyday use but the lack of aesthetics, which will remain unless a tooth-colored material with the same properties as the SSC is developed.

### 4.4. Recommendation for Clinicians

SSCs appear to be superior in success and survival in comparison to ZOC for the post-pulpotomy restoration of deeply carious primary molars and should be used primarily.

Despite the aesthetic disadvantage, SSCs are highly accepted and show a wider range of advantages over ZOCs, which are more prone to failure as they are more technique sensitive and costly. However, generalizability of the findings regarding ZOCs may be regarded as limited as only one dentist performed the treatment and one may argue that a more experienced/excellent operator could achieve better outcomes, while results for SSCs with about 90% were in line with the literature [6].

### 4.5. Recommendation for Future Research

RCTs comparing SSC and ZOC would be interesting for future investigations to minimize the impact of case selection and socio-economic factors. Though this study design has a high level of scientific evidence, the implementation in real life may differ and portray different outcomes. This is especially crucial as usually pulpotomy and a following restoration are not always performed under “perfect study conditions” like in RCTs in university clinics.

Therefore, further long-term prospective studies under real-life conditions in other countries and settings with a longer follow-up period would strengthen the conclusions drawn from this research.

## 5. Conclusions

Preformed stainless steel and zirconium oxide crowns placed on pulpotomized deciduous molars with 15.5% ferrous sulphate solution to stop bleeding and zinc oxide eugenol cement as a wound dressing have a good clinical survival rate. Nonetheless, the zirconium oxide crown was not an equivalent alternative for the restoration of pulpotomized deciduous molars in this real-world setting, as the stainless steel crown has significantly higher clinical survival rates, is less expensive, easier to apply, and, at the same time, is also very well accepted. This must be carefully weighed against the slightly higher aesthetic acceptance of the zirconia crown when making a clinical decision regarding the restoration.

## Figures and Tables

**Table 1:** General baseline characteristics of the study sample at patient level.

Category	Factor	Total (n = 100)
Gender	Male Female	57 (57%) 43 (43%)
Health insurance	Statutory Private	92 (92%) 8 (8%)
Place of residence Living distance from dental office	0–20 km radius 20–50 km radius >50 km radius	70 (70%) 26 (26%) 4 (4%)
Age group in years	2–5 yrs 6–8 yrs 9–10 yrs	48 (48%) 41 (41%) 11 (11%)
Baseline age	Mean in years (±SD)	5.29 (±2.12)
Baseline caries experience in primary teeth	Mean dmft (±SD)	7.08 (±3.18)
Baseline caries experience in permanent teeth	Mean DMFT (±SD)	0.10 (±0.38)

**Table 2:** Baseline characteristics of the study sample on tooth level differentiated for stainless steel crown and zirconium oxide crown.

Category	Factor	SSC (n = 203, 74.6%)	ZOC (n = 69, 25.4%)	Total (n = 272, 100%)	Stat Sign. Chi^2^ Test^-^
Clinical setting of the treatment	Chairside/LA N_2_O-sedation DGA	5 44 154	0 1 68	5 (1.8%) 45 (16.5%) 222 (81.6%)	***p* < 0.005**
Jaw	Maxillary Mandibular	69 88	12 65	81 (34.7%) 153 (65.3%)	*p* = 0.210
Molars	First primary molar Second primary molar	92 111	34 35	126 (46.3%) 146 (53.7%)	*p* = 0.569
ICDAS	4 5 6	32 164 7	8 61 0	40 (14.7%) 225 (82.7%) 7 (2.6%)	*p* = 0.187
Mean follow-up time in months (±SD)		28.7 (±11.3)	26.7 (±10.1)	28.2 (±11.0)	*p* = 0.192

SSC: Stainless steel crown; ZOC: Zirconium oxide crown; ICDAS: International Caries Detection and Assessment System (caries classification); SD: standard deviation; LA: Local anesthesia; N_2_O-sedation: Nitrous oxide sedation; DGA: Dental General Anesthesia; *p* < 0.05 in bold referring to statistically significantly different.

**Table 3:** Mean estimated survival times in months of stainless steel and zirconium oxide crowns.

Category	Mean Survival Time (Months)			Log-Rank (Mantel–Cox)	
Restoration	Estimate	Std. Error	95% CI	Chi^2^-Test	*p* Value
SSC	31.4	0.46	30.5–32.4	7.63	**0.006**
ZOC	23.4	0.69	22.0–24.7		
Overall	30.6	0.48	29.7–31.5		

SSC: Stainless steel crown; ZOC: Zirconium oxide crown; *p* < 0.05 in bold referring to statistically significantly different.

**Table 4:** Evaluation of primary outcome (surviving/failure) according to post-pulpotomy restoration (stainless steel crowns vs. zirconium oxide crowns).

Evaluation *	Total (n = 272, 100%)	SSC (n = 203, 74.6%)	ZOC (n = 69, 25.4%)	Stat. Sign Chi^2^-Test
Surviving	220 (80.9%)	180 (88.7%)	40 (58.0%)	***p* < 0.005**
Minor failure	19 (7.0%)	10 (4.9%)	9 (13.0%)	
Major failure	33 (12.1%)	13 (6.4%)	20 (29.0%)	
Occurrence of a Fistula	13 (39.4%)	2 (15.4%)	11 (55.0%)	***p* = 0.018** for major failure
Swelling and Abscess	15 (45.4%)	10 (77.0%)	5 (25.0%)	
Fracture of the crown and abutment	5 (15.2%)	1 (7.6%)	4 (20.0%)	

SSC: Stainless steel crown; ZOC: Zirconium oxide crown; * Definitions see methods. Percentages shown per column adding up to 100%; For subdivision within major failure percentages shown per column adding up also to 100%; *p* < 0.05 in bold referring to statistically significantly different.

**Table 5:** Evaluation of primary outcome (surviving vs. failure) according to post-pulpotomy restoration (stainless steel vs. zirconium oxide crowns) differentiated by clinical setting of the treatment.

Category	SSC (n = 203)		ZOC (n = 69)	
Clinical Setting & Treatment Modality	Surviving	Failed	Surviving	Failed
LA	5 (2.6%)	0	0	0
N2O-sedation + LA	37 (19.5%)	7 (53.8%)	1 (2.0%)	0
DGA	148 (77.9%)	6 (46.2%)	48 (98%)	20 (100%)
Total	190 (100%)	13 (100%)	(100%)	20 (100%)

SSC: Stainless steel crown; ZOC: Zirconium oxide crown; LA: Local Anesthesia; N_2_O-sedation: Nitrous oxide sedation; DGA: Dental General Anesthesia.

## Data Availability

Data supporting reported results can be provided by the authors upon justified request. Some of the data were presented at the 71st ORCA Congress in Heraklion, Crete, Greece, 3–6 July 2024. The abstract (Nr. 155) is available online free of charge under https://karger.com/cre/article-pdf/58/3/184/4254291/000539768.pdf (accessed on 23 January 2025) [26].
